# Telescoping Allograft Prosthetic Composite (APC) Reconstruction of the Femur Following Revision Arthroplasty for Neglected Developmental Dysplasia of the Hip (DDH)

**DOI:** 10.7759/cureus.39925

**Published:** 2023-06-03

**Authors:** Jin Chuan Yuen, Hee Nee Pang, Ren Yi Kow

**Affiliations:** 1 Orthopaedics, Hospital Tengku Ampuan Afzan, Kuantan, MYS; 2 Orthopedics, Singapore General Hospital, Singapore, SGP; 3 Orthopaedics, Traumatology & Rehabilitation, International Islamic University Malaysia, Kuantan, MYS

**Keywords:** developmental dysplasia of the hip, bone allograft, allograft prosthesis, limb lengthening, limb reconstruction, reconstruction hip and knee surgery, hip reconstruction

## Abstract

Although uncommon, neglected developmental dysplasia of the hip (DDH) poses a technically demanding problem for treating surgeons. Due to the congenital malformation of the native hip joint and distortion of the surrounding soft tissue, addressing limb-length discrepancy is intricate. Despite detailed planning and meticulous soft tissue handling, complications can be difficult to avoid in these patients even under experienced hands. In this case report, we present a 73-year-old lady with neglected DDH who had undergone initial total hip arthroplasty and subsequent revision surgery that failed due to aseptic loosening. Due to limited length in the distal femur, we used a telescoping allograft prosthetic composite (APC) to provide adequate length to the native distal femur during revision with proximal femur fixation. This technique can help avoid the need for total femur replacement (TFR) surgery, which is more invasive and may require tibia replacement.

## Introduction

Developmental dysplasia of the hip (DDH) is a congenital condition in which the hip joint of a newborn baby is dislocated or unstable. DDH predisposes patients to early osteoarthritis and limited ambulatory status if not diagnosed and treated promptly. The incidence of DDH is approximately 1 in 1,000 live births [1]. As clinicians become more aware of this condition and with the identification of risk factors and advancements in screening tools, more babies with DDH are being detected and managed early [1,2]. Nevertheless, there are some patients who present in adulthood with neglected DDH [1].

A neglected DDH is a multifaceted disease that requires careful consideration by the treating clinician [1,2]. First, due to development predicaments that prevent the normal formation of hip joint, the morphology of the acetabulum and proximal femur is characterized by inadequate coverage of the femoral head, a shallow and anteverted acetabulum, and limited anterior acetabulum wall. In addition to bony deformity, the soft tissues surrounding a dysplastic hip joint are relatively shortened and atrophic, resulting in excessive adduction and flexion contractures of the affected limb [1]. As a result of the deformed hip morphology and distorted soft tissues, correcting limb length discrepancy in a patient with neglected DDH can be challenging.

Arthroplasty surgery in older age is the treatment of choice for patients with neglected DDH and early osteoarthritis of the hip [3-6]. Nonetheless, detail-planning is essential to ensure optimal outcomes, taking into consideration of the distorted bony anatomy and soft tissue contractures around the hip. In cases where the implant fails, revision surgery is often more challenging than the index surgery, especially if there is limited bony stock that enable stem integration to hold. For such cases, megaprosthesis, such as total femur replacement (TFR) endoprosthesis, has been proposed. However, TFR requires extensive surgical incision and replacement of the tibia in the same setting. Therefore, in this case study, we present a 73-year-old lady with neglected DDH whose initial total hip arthroplasty failed, and she was treated with an allograft prosthesis composite (APC) to avoid TFR.

## Case presentation

**Figure 1:**
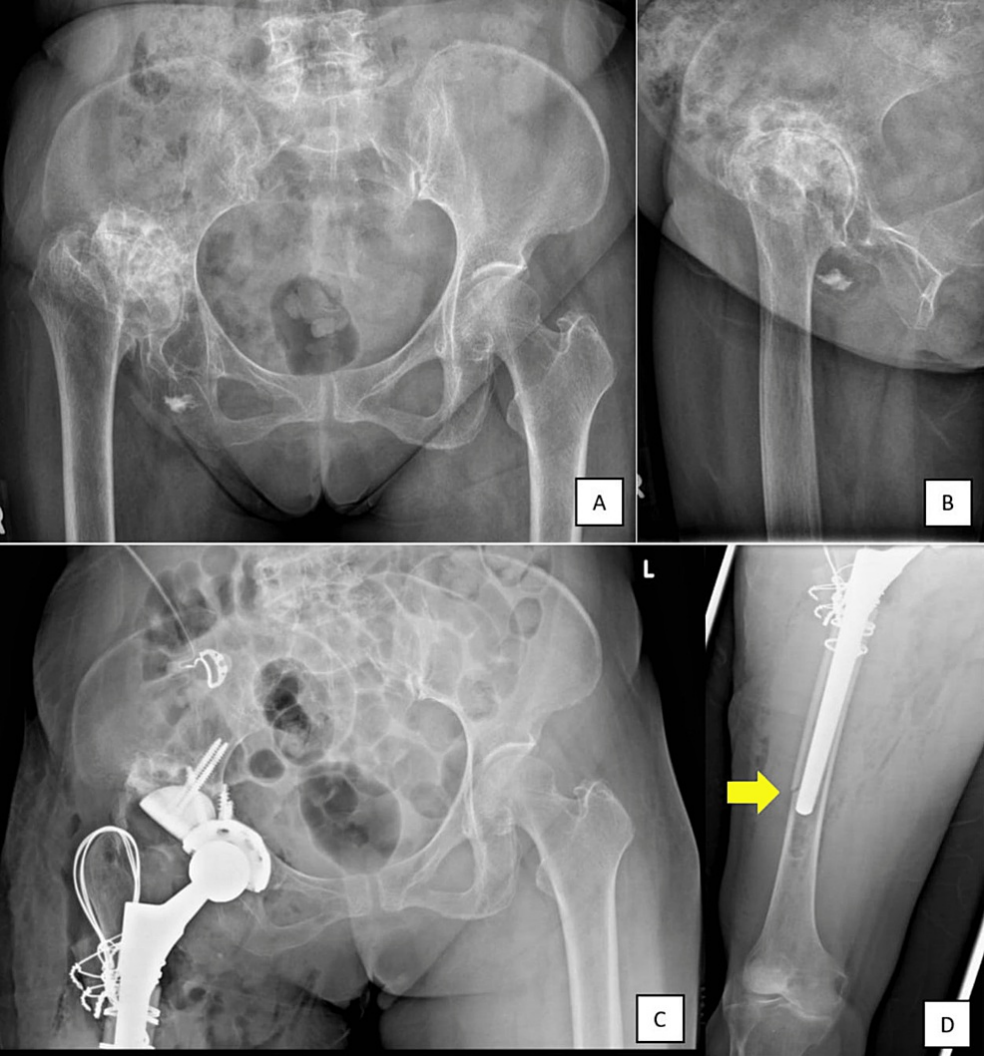
(A) Plain radiograph of the pelvic prior to the surgery; (B) Plain radiograph of the right hip in lateral view; (C) Plain radiograph of the pelvic after the index right total hip replacement (THR). A cementless stem was inserted, and the superolateral dysplastic pseudoacetabulum was reconstructed with acetabulum cup and augmented with trabecular metal; (D) A femur iatrogenic fracture occured during the index total hip reconstruction (yellow arrow).

A 73-year-old female presented with secondary osteoarthritis of the right hip, which was caused by neglected development dysplasia of the hip (Figure 1A and 1B). She underwent right total hip replacement (THR) with cementless stem insertion, and the superolateral dysplastic pseudoacetabulum was reconstructed with acetabulum cup and augmented with trabecular metal (Figure 1C). The right limb shortening of 4 cm was addressed with bone grafting at the area between greater trochanter and femoral shaft using a Wagner SL revision stem with cerclage wiring of the greater trochanter and femoral shaft (Figure 1C and 1D). However, there was a femur iatrogenic fracture during the index total hip reconstruction, which was treated conservatively (Figure 1D). Three months after the index right hip surgery, the fracture appeared to be healing with signs of callus formation, but radiolucency was detected around the tip of the stem. The patient was allowed to ambulate with partial weight bearing three months after the surgery, and was able to ambulate with a walking frame six months after surgery.

**Figure 2:**
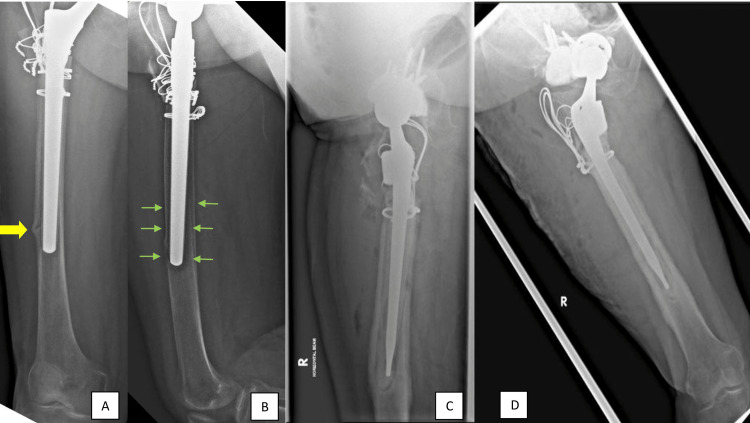
(A) Three months after the index right hip surgery, the fracture appeared to be healing with signs of callus formation (yellow arrow); (B) Two years after the surgery, plain radiograph of the right hip showed well-united fracture but also revealed peri-implant radiolucency (green arrows), suggestive of stem loosening; (C) Plain radiograph of the right hip in lateral view after the patient underwent revision surgery where a cemented stem was inserted with a revision/calcar hip system and a shortening osteotomy; (D) Plain radiograph of the right hip in anteroposterior view.

She was asymptomatic until two years after the initial right hip surgery when she presented with gradually increasing pain in the right hip that incapacitated her (Figure 2A). Plain radiograph of the right hip showed well-united fracture but also revealed peri-implant radiolucency, suggestive of stem loosening (Figure 2B). Her biochemical parameters were unremarkable, and she underwent revision surgery for aseptic loosening of the right THR stem. The revision surgery involved a cemented stem revision with a revision/calcar hip system and a shortening osteotomy (Figure 2C and 2D). Unfortunately, the revision surgery was complicated by periprosthetic joint infection (PJI), and the patient developed surgical site erythema with raised septic parameters. Subsequently, she underwent right hip wound debridement and washout for the PJI, and intra-operative samples grew *Staphylococcus capitis*. The patient was treated with a course of intravenous piperacillin-tazobactam for two weeks and oral clindamycin for the next three months based on the culture and sensitivity results. Following treatment, the patient made a full recovery and was able to ambulate with a walking frame three months after the surgery.

**Figure 3:**
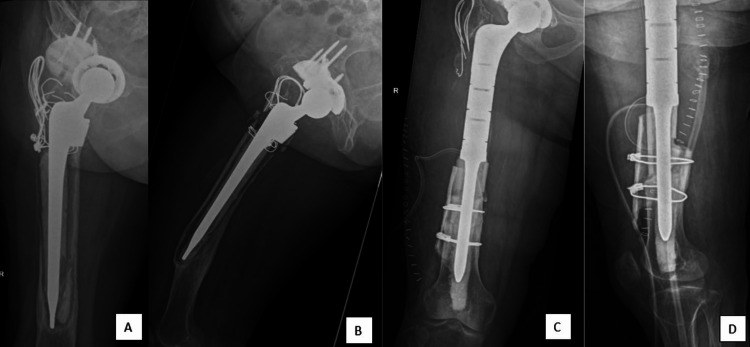
(A) Plain radiograph of the right hip in anteroposterior view revealed loosening of the implant with the tip of the stem spanning 7 cm from the knee joint; (B) Lateral view of the right hip plain radiograph; (C) Plain radiograph in anteroposterior view after revision with allograft prosthesis composite (APC). A size-12 stem was used in this case with a length of 125 mm; (D) Lateral view of the plain radiograph.

**Figure 4:**
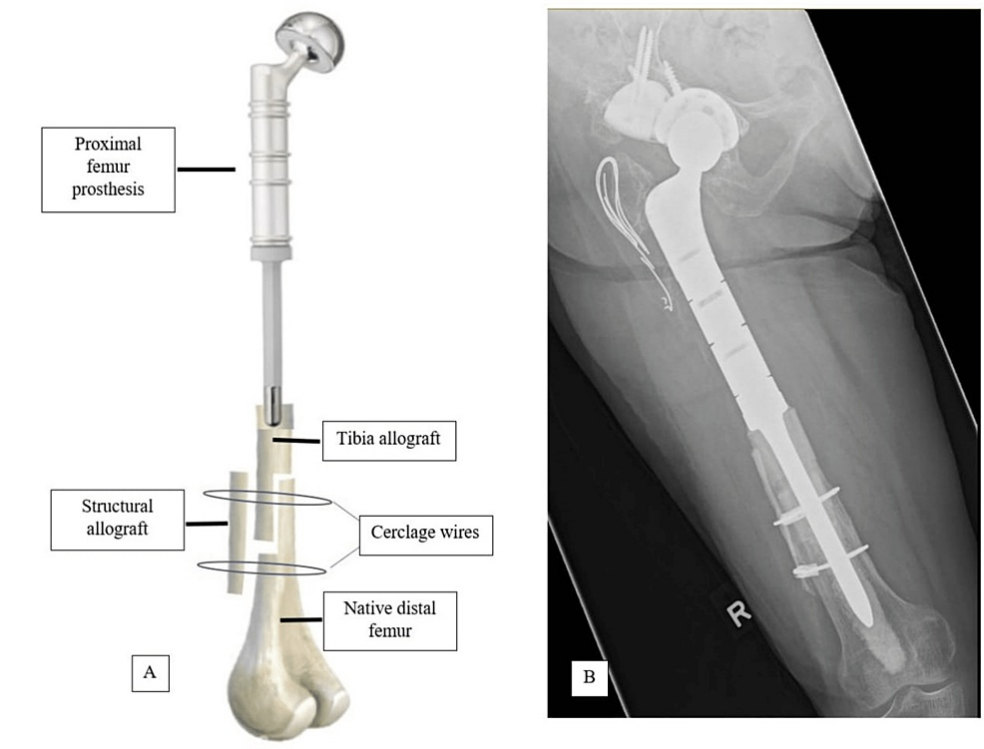
(A) The breakdown of the allograft prosthesis composite (APC) construct. Cerclage cable with crimps are being used as the cerclage wires; (B) Plain radiograph of the right femur revealed a well-incorporated APC.

Three years after the revision surgery (five years after the index surgery), she presented with increasing pain in her right hip, which affected her quality of life. A plain radiograph of the right hip revealed loosening of the implant with the tip of the stem spanning 7 cm from the knee joint (Figure 3A and 3B). She was diagnosed with aseptic loosening of the right hip implant since her septic parameters were normal. In order to avoid total femur megaprosthesis, the revision was performed with APC since the length of the original distal femur was inadequate for proximal femur prosthetic fixation (Figure 3C and 3D). The breakdown of the APC construct is illustrated in figure 4A. The patient recovered well (Figure 4B). One year after the revision surgery, she was able to ambulate with a walking frame without any pain. The patient was satisfied with the revision surgery. 

## Discussion

The difficulty of managing a neglected DDH is illustrated in this case. The index surgery was complicated with iatrogenic fracture of the femur shaft due to dysplastic bone of the femur. Although the fracture was subsequently healed with conservative management, micromotion present at the femoral stem leads to aseptic loosening. Even though the first revision surgery was complicated with PJI, she recovered well with debridement, antibiotic and implant retention (DAIR).

Three years after the revision surgery (five years after the index surgery), she presented with another episode of aseptic loosening. At this stage, the treatment options include the use of long revision stems, APCs, and proximal femur replacement (PFR) [5-7]. However, these options were not viable owing to the poor bone and tissue conditions in this patient. Even more invasive procedures, such as TFR and resection arthroplasty, had been proposed [6,7]. In the revision surgery, the new distal stem must be of sufficient length to achieve rigid fixation into the remaining diaphyseal femur fragment. In general, the stem must be embedded in the intramedullary by at least 100 mm for both cemented or uncemented fixation [5-8]. Thus, stem insertion is not feasible in this case where only 70 mm of the native distal femur remains. The native distal femur is further shortened during the revision surgery where the bone surrounding tip of the previous implant is removed. Compressive osteointegration stems, custom-made short medullary stems, stems with cross-fixation pins, and extracortical plates have been used to reconstruct femurs with a short proximal segment, but these techniques do not lengthen the native distal femur [8,9]. Similarly, modified endoprosthesis stems are not feasible as they do not add to the bone stock of the femur and are predisposed to bone resorption and subsequent stem loosening that necessitate a TFR later [10]. Due to these reasons, PFR implant was not feasible in this patient.

Although TFR is a viable option, it is more invasive and requires replacement of the tibia component. Therefore, to build the length of the femur, a tibial allograft is used to extend the length of the distal femur and enable the fixation of a PFR. Most cases published on APCs involve reconstructing the proximal part of the long bone using a proximal femoral allograft fixed with a long stem. However, in our case, the remaining length of the distal femur did not allow for this approach. We adopted the technique by Healey et al., in which a telescopic allograft reconstruction is used to reconstruct the diaphysis in limb salvage surgeries and to augment the length of the native bone [11]. By adding multiple bone allografts, the length of the limb can be reconstructed, and the allograft provide adequate biomechanical support for the APC in the revision surgery [12].

## Conclusions

Arthroplasty surgery is technically difficult to perform in a patient with neglected DDH. Detailed planning, in which both bony and soft tissues are carefully evaluated, is essential in minimizing complications. In a situation where PFR is not feasible due to inadequate native distal femur bone stock, a telescoping APC can be used to avoid TFR. 
